# The causal effects of childhood sunburn occasions on melanoma: A univariable and multivariable Mendelian randomization study

**DOI:** 10.1515/med-2024-1078

**Published:** 2024-11-29

**Authors:** Wei Sun, Huihui Sun, Chong Yu

**Affiliations:** Department of Radiation Oncology, The Third Affiliated Hospital of Nanjing Medical University, Changzhou Second People’s Hospital, Changzhou Medical Center, Nanjing Medical University, Changzhou, Jiangsu, 213000, China; Department of Radiation Oncology, The Third Affiliated Hospital of Soochow University, Changzhou, 213003, China; Department of Radiation Oncology, Changshu Hospital Affiliated to Soochow University, Changshu No. 1 People’s Hospital, Changshu, 215500, Jiangsu, China

**Keywords:** childhood sunburn occasions, melanoma, onset risk, Mendelian randomization study

## Abstract

Observational studies have shown an association between childhood sunburn occasions (CSOs) and melanoma *in situ* (MIS). However, these studies have shown contradictory results. Here, we used a two-sample Mendelian randomization (MR) method to make a causal inference between CSOs and melanoma at the genetic level. Based on the publicly available genome-wide association study summary data, including childhood sunburn (*n* = 346,955) and MIS (*n* = 218,792), the inverse-variance weighted (IVW) method of the random effects model was used, supplemented by the MR-Egger method, the weighted median method, and the weighted mode method. IVW results showed a 2.58-fold increased risk of melanoma development for each standard deviation increase in CSOs (odds ratio [OR] = 3.58; 95% confidence interval [CI]: 1.68–7.64; *P* = 1.00 × 10^−3^), with the MR-Egger (OR = 4.76, 95% CI: 1.65–13.75, *P* = 5.60 × 10^−3^), weighted median (OR = 4.89, 95% CI: 1.62–14.76, *P* = 4.90 × 10^−3^), and weighted mode (OR = 6.26, 95% CI: 2.49–15.77, *P* = 3.00 × 10^−4^) supporting the results. Furthermore, both the funnel plot and the MR-Egger intercepts showed the absence of directional pleiotropy between childhood sunburn and MIS. Our study confirmed that CSOs increase the risk of melanoma development.

## 1 Introduction

The global burden of cancer is currently high, posing a great public health challenge. Additionally, the incidence and mortality of melanoma, a highly aggressive and fatal malignant tumor, are increasing every year, with the age of onset tending to be younger [1]. Therefore, it is crucial to obtain an in-depth understanding of melanoma’s etiology and risk factors and accordingly establish a reasonable and comprehensive hierarchical prevention and management system.

Melanoma is a result of the interaction of genetic and environmental factors [2]. In recent years, an increase in outdoor activities (e.g., surfing, sunbathing, camping, and cycling) and the popularity of the bodybuilding culture have led to an overload of ultraviolet light on the human body [3–5]. Moreover, prolonged exposure to intense light and inadequate sun protection measures (e.g., applying sunscreen, wearing protective clothing, and seeking shade) often cause acute skin damage, namely sunburn [6].

Researchers have previously evaluated the association between childhood sunburn occasions (CSOs) and melanoma using case–control or cohort studies; however, the findings have been controversial. For example, a cohort study from Norway found that childhood sunburn increased the risk of developing melanoma [7]. Another study, by Olsen et al. [8], indicated that having more than 50 sunburn experiences during childhood or adolescence can significantly increase the risk of melanoma development. However, in a multicenter case-control study initiated in seven European countries, researchers found that the risk of melanoma development was associated with the frequency of sunburns but not related to the age during the sunburn occasions [9]. This difference in findings may be due to the vulnerability of observational epidemiological studies to potential confounders, reverse causality, and measurement error [10–12]. Therefore, clarifying the real relationship between CSOs and melanoma is important for the better prevention and control of melanoma. Randomized controlled trials (RCTs) are regarded as the “gold standard” for causal inference. The randomized grouping of study subjects increases comparability between groups and effectively addresses selection bias and confounding issues. Nevertheless, RCTs are difficult to conduct in many medical studies owing to their high cost, cumbersome implementation, and poor subject compliance, in addition to the inappropriate use of placebo, inappropriate selection of control group measures, or exposure of subjects to certain harmful or pathogenic risk factors, which are contrary to medical ethics [13]. Therefore, there is an urgent need for a novel and robust epidemiological research tool that can both emulate the RCT in accurately inferring the causal relationship between sunburn and melanoma in childhood and overcome the drawbacks of observational studies.

With the recent advances in multi-omics technologies and the accumulation of data from genome-wide association studies (GWAS), Mendelian randomization (MR) has become a widely used tool for causal inference studies of diseases [14,15]. The MR method uses genetic variants that are strongly associated with exposure factors as instrumental variables (IVs) and the risk of developing complex diseases as outcomes to infer causal effects between exposure factors and outcomes [16,17]. Since gamete formation follows Mendel’s second law of inheritance, which states that “parental alleles are randomly assigned to offspring,” genetic variation is not influenced by traditional confounding factors such as other population-specific factors, environmental exposures, and socioeconomic status. Moreover, as genetic variation is inherited from parents and remains constant after birth, its association with outcomes has event sequence rationality. Therefore, MR can not only effectively avoid the interference of potential confounding factors and reverse causation but also save time, human, and material resources to a great extent and be more cost-effective [18–21]. In the field of oncology, MR analysis is mainly used for etiological studies, including but not limited to the influence of environment, traits, etc., on the occurrence, development, and regression of tumors, providing new ideas for the treatment and prognosis of tumors. Thus, MR is an ideal method to explore the causal relationship between CSOs and melanoma.

Interestingly, previous MR studies have suggested a possible causal relationship between childhood sunburn and melanoma, but they both have strengths and limitations [22–24]. The current study assessed the causal effect of CSOs and melanoma risk at the genetic level using the two-sample MR (TSMR) method based on large GWAS summary data.

## 2 Materials and methods

### 2.1 Research design

**Figure 1:**
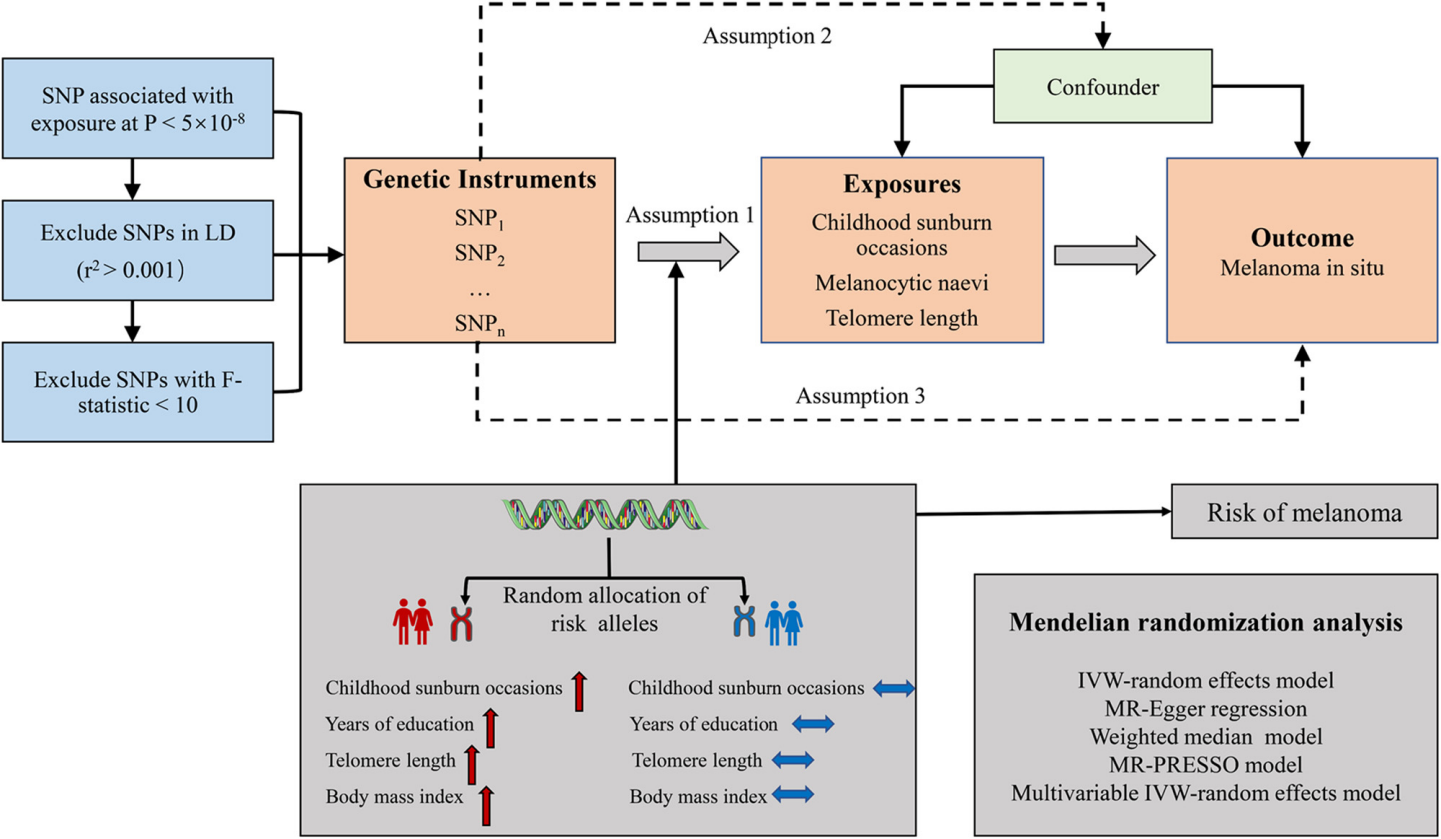
Overview and assumptions of the MR study design.

The TSMR research design is shown in Figure 1. To obtain unbiased estimates, single-nucleotide polymorphism (SNP) loci as IVs were considered to satisfy three core assumptions [25, 26]: (1) IVs are strongly correlated with exposure factors (relevance assumption); (2) IVs are independent of any confounding factors affecting the “risk of outbreak-outcome” (independent assumption); and (3) IVs can have effects on outcomes only through exposure factors and not through other pathways (exclusion restriction assumption).

### 2.2 Data sources of CSOs

The exposure-related GWAS and CSOs were obtained from the UK Biobank. Since its establishment in 2006, the database has recruited 500,000 participants across the United Kingdom and obtained genetic, life environment, and health data based on a large population sample through various forms of biological sample collection and questionnaires [27, 28]. Furthermore, the data related to CSOs were collected from participants who filled out their questionnaires using Touchscreen. Participants were asked, “Before the age of 15, how many times did you suffer sunburn that was painful for at least 2 days or caused blistering?” The participant was excluded if the answer was <0 or >999; if the answer was >20, then the participant was included after the response was confirmed a second time; other options were “−1” or “−3,” representing “do not know” or “unwilling to answer,” respectively. Finally, 346,955 participants of European origin were included.

### 2.3 Melanoma data sources

GWAS summary-level genetic data for melanoma were obtained from the FinnGen Alliance R5 release (https://r5.finngen.fi). The phenotype of “melanoma *in situ* (MIS)” was selected for this study, and this dataset included 218,792 Finnish individuals, including 393 cases and 218,399 controls, after removing excess heterozygosity (±4 standard deviation [SD]), high genotype deletions (>5%), gender ambiguity, and individuals of non-Finnish ancestry. All genetic association effect sizes were calculated by logistic regression and adjusted for age, sex, ten principal genetic components, and genotyping batches [29].

### 2.4 IV screening and validation

In this study, the SNP loci associated with CSOs were screened according to the following criteria: (i) the association of SNP loci with CSOs reached a whole-genome significance level, namely *P* < 5.0 × 10^−8^, satisfying the core assumption (1); (ii) the linkage disequilibrium (LD) coefficient *R* ^2^ was set to 0.001, and the width of the LD region was 10,000 kb, and the SNP loci with LD were removed to ensure that each locus was independent of each other; and (iii) the *F*-statistic was calculated for each SNP locus, excluding weak IVs (*F* < 10) [25]. The *F*-statistic was calculated using the following equation:

(1) \[F=\frac{N-K-1}{K}\times \frac{{R}^{2}}{1-{R}^{2}}.]\]

Since *R* ^2^ is not directly available, it was calculated with the help of the following equation:

(2) \[{R}^{2}=2\times (1-{\mathrm{MAF}})\times {\mathrm{MAF}}\times {\left(\frac{\beta }{{\mathrm{SD}}}\right)}^{2}.]\]

In the above equations, *N* is the number of samples in the exposed data set, *K* is the number of SNPs, MAF is the minor allele frequency, *β* is the effective value of SNP on exposure, and SD is the standard deviation.

Initially, 62 CSOs associated with SNP loci were screened. The rsid, effect allele, reference allele, MAF,the effect value of the loci associated with CSOs, standard error, and *P*-value of the above loci were extracted. Then, the basic information of the above CSO-associated SNP loci and the effect values, standard errors, and *P*-values of the SNP loci associated with melanoma were extracted from the melanoma database. To satisfy assumption (3), if the association of an SNP locus with melanoma reached *P* < 5.0 × 10^−8^, the SNP locus was not included in the analysis as an IV. In addition, SNP loci with missing allelic information and those that were themselves palindromic sequences were excluded. Finally, 59 SNPs were included as IVs for subsequent analysis.

### 2.5 Univariate MR analysis

To assess the causal effect between CSOs and melanoma, inverse variance weighted (IVW) was used as the subject analysis method. When all SNPs are valid IVs, the overall bias of IVW is zero. If invalid instruments are present, biased estimates are obtained [25]. Therefore, to avoid biased results due to multi-effectiveness, we also used the weighted median, weighted mode, MR-Egger method, and MR Pleiotropy RESidual Sum and Outlier (MR-PRESSO) to conduct sensitivity analysis on the causality of IVW estimates and to compare the variability of the results obtained by the various methods to assess the robustness of the study results with regard to the assumption of multiplicity [30–32]. The estimates obtained using the different methods were consistent, suggesting that the potential bias introduced by the pleiotropic IVs was effectively corrected, further indicating that the conclusions drawn by the IVW method are reliable and robust.

### 2.6 Sensitivity analysis

To avoid the confounding effect of pleiotropism on causal associations, the following sensitivity analysis methods were used. First, Cochran’s *Q* test was used to test the heterogeneity of SNP loci [31]. A *P* > 0.05 was considered to suggest that there is no heterogeneity; in other words, the core assumptions (2) and (3) are satisfied, at which point the individual SNP locus is proportionally associated with the strength of CSOs and melanoma. Second, the MR-Egger intercept test and MR-PRESSO method were used to assess the horizontal pleiotropy of the SNPs included in the analysis [33, 34]. When using the MR-Egger intercept test, if the intercept was not significant (*P* > 0.05), it was considered to indicate that the SNPs included in the analysis are not pleiotropic; that is, they satisfy the core assumption (3). In contrast, the MR-PRESSO method can both detect the horizontal pleiotropy of SNPs included in the analysis and identify and reject outliers and thus correct for horizontal pleiotropy. Third, the SNP loci associated with confounding factors were excluded using the online webpage PhenoScanner_v2_ (http://www.phenoscanner.medschl.cam.ac.uk) to query the disease or phenotype associated with each SNP locus [35]. If an SNP locus was associated with possible confounding factors, it was excluded, and MR analysis was performed again to verify the core assumption (2). Fourth, a leave-one-out analysis was conducted wherein SNPs were removed one by one, and the causal effects were recalculated by the IVW method using the remaining SNPs. Forest plots were drawn to judge the degree of influence of individual SNPs on the causal effect estimation [36]. If the result changed significantly after eliminating an SNP, it suggested that the SNP is a potential outlier and is considered for elimination. Finally, a Funnel plot was used for a visual check of symmetry to determine whether there were obvious outliers.

### 2.7 Multivariate MR analysis

Multivariate MR analysis is an extension of univariate MR analysis. It integrates SNP associated with multiple exposures into the model so that the direct effect of a given exposure on the outcome can be assessed after controlling for other exposures [37]. Previous studies have shown that telomere length [38], body mass index (BMI) [39], and years of education [40] are associated with melanoma development. To test whether potential risk factors influenced the findings, we broadened the exposure factors included in the study to include telomere length, BMI, and years of education.

### 2.8 Statistical software

This study used the R package of R-4.1.0 software (“TwoSampleMR,” “MRPRESSO,” “dplyr,” and “forestplot”) for analysis and plotting. Bilateral *P* < 0.05 was considered a statistically significant difference.


**Ethical approval:** The manuscript does not contain clinical studies or patient data. Our study is based on the large-scale GWAS datasets, and not the individual-level data, and no additional ethical approval was applicable.

## 3 Results

### 3.1 Tool variable details

**Figure 2:**
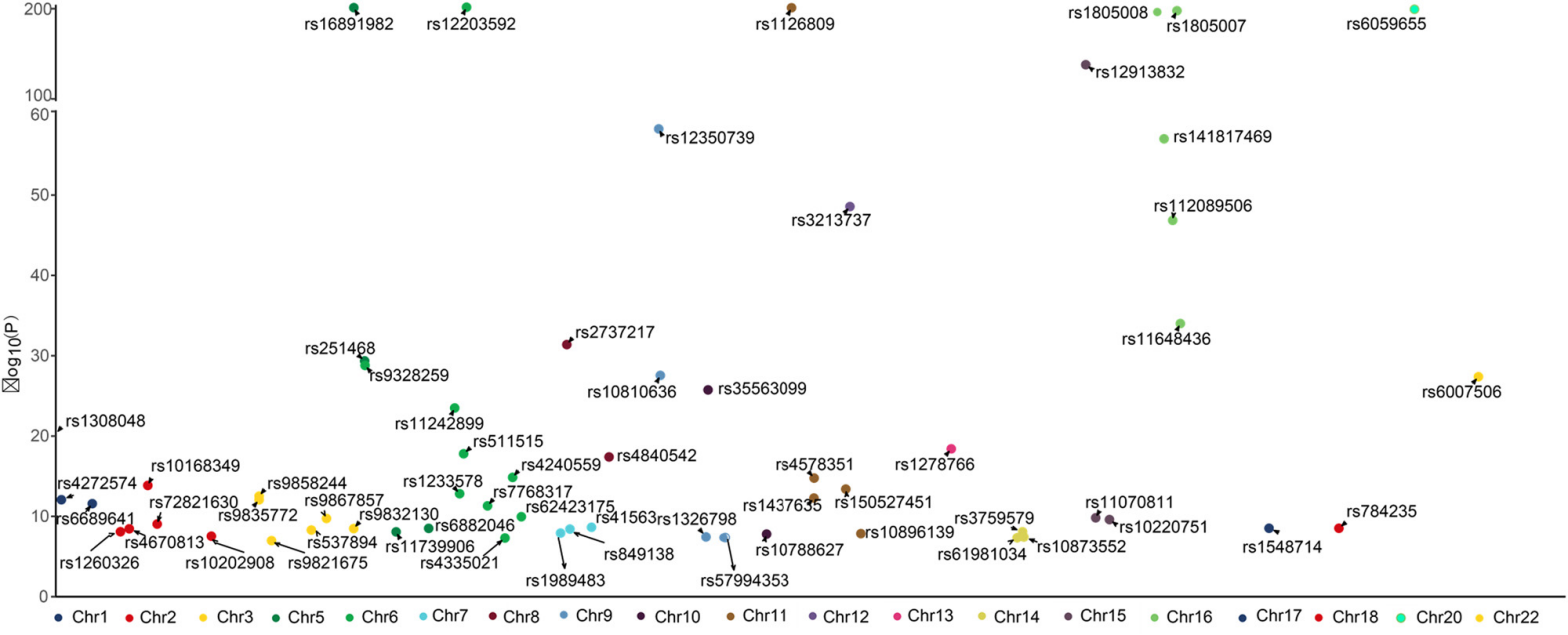
Manhattan plot of 59 SNPs identified as IVs from exposure dataset.

After rigorous screening and validation, we finally included 59 SNPs as IVs. The Manhattan plots of these 59 SNPs are shown in Figure 2. Table 1 shows the basic information of these SNPs, including their location on the chromosome, effect allele, other alleles, the effect allele frequency, *P*-values associated with CSOs, and the *F* statistic. The *F* statistic for each SNP was greater than 10, suggesting that weak IVs did not influence this study.

**Table 1:** Characteristic of SNPs extracted from exposure (childhood sunburn occasions) GWAS statistical summary data

SNP	Chromosome: Position	EA	OA	EAF	*F*-statistic	Association with exposure	
						SE	*P*-value
rs4272574	1:73661205	T	C	0.478288	25.54824371	0.00212901	8.4004 × 10^−13^
rs1308048	1:66888542	C	T	0.419538	43.06382755	0.00216433	5.30029 × 10^−21^
rs6689641	1:110720400	G	A	0.542588	24.30509747	0.00213425	2.60016 × 10^−12^
rs10168349	2:46360907	C	G	0.335704	26.39488164	0.00225444	1.39991 × 10^−14^
rs1260326	2:27730940	C	T	0.603989	15.90840137	0.00217252	8.10009 × 10^−9^
rs72821630	2:63696212	T	C	0.251407	14.12595433	0.00245522	8.99995 × 10^−10^
rs4670813	2:38317710	A	G	0.470775	17.396246	0.00214417	3.50002 × 10^−9^
rs10202908	2:169378292	T	C	0.674884	13.51532079	0.00227125	2.90001 × 10^−8^
rs9835772	3:85766025	T	A	0.243745	18.85371494	0.00247742	8.60003 × 10^−13^
rs537894	3:138348595	A	G	0.548976	16.92221943	0.00217696	0.000000005
rs9821675	3:49902544	G	A	0.50527	16.04236262	0.00212501	0.000000015
rs9867857	3:156491160	T	C	0.48916	20.34205035	0.00213562	1.79999 × 10^−10^
rs9832130	3:189194752	A	G	0.58068	17.05000098	0.00216232	3.29997 × 10^−9^
rs9858244	3:85787399	A	G	0.214182	17.91747205	0.00259673	2.99985 × 10^−13^
rs16891982	5:33951693	G	C	0.970879	77.04680338	0.00620874	1 × 10^−200^
rs11739906	5:59019359	C	A	0.327463	14.6190973	0.00226464	8.40001 × 10^−9^
rs251468	5:149194485	T	C	0.248353	48.49518624	0.00247434	4.40048 × 10^−30^
rs6882046	5:87968864	G	A	0.26793	13.77104938	0.00242202	3.09999 × 10^−9^
rs9328259	6:508972	A	C	0.718185	51.54674607	0.00237012	1.59993 × 10^−29^
rs1233578	6:28712247	G	A	0.176418	15.85660412	0.00278697	1.50003 × 10^−13^
rs7768317	6:41922220	T	C	0.248878	17.84587021	0.00246523	4.90004 × 10^−12^
rs4335021	6:32386619	C	T	0.59972	14.28623501	0.00216154	4.90004 × 10^−8^
rs12203592	6:396321	T	C	0.21907	1261.727892	0.00252627	1 × 10^−200^
rs511515	6:33541507	G	A	0.70089	32.34252089	0.00232118	1.59993 × 10^−18^
rs11242899	6:460302	A	G	0.266333	40.28577146	0.0024111	3.19963 × 10^−24^
rs4240559	6:98437775	C	T	0.557906	31.4415833	0.00214258	1.39991 × 10^−15^
rs41563	7:104852654	A	G	0.349781	16.65217657	0.00223217	1.40001 × 10^−9^
rs849138	7:28177338	A	G	0.507025	17.36330987	0.00213095	3.79997 × 10^−9^
rs1989483	7:16942661	G	A	0.389054	15.48272013	0.00218968	0.000000012
rs4840542	8:10944809	T	G	0.505804	37.65079257	0.00213294	4.00037 × 10^−18^
rs2737217	8:116630311	G	A	0.563309	68.47860306	0.00215785	4.10015 × 10^−32^
rs10810636	9:16799109	G	A	0.760343	44.33563678	0.00249423	2.80027 × 10^−28^
rs12350739	9:16885017	A	G	0.606787	125.1419914	0.00219034	5.79963 × 10^−59^
rs57994353	9:139356987	C	T	0.299074	12.5853042	0.00232272	4.30002 × 10^−8^
rs1326798	9:12722227	G	C	0.620628	14.29109844	0.00219225	3.59998 × 10^−8^
rs35563099	10:119572403	T	C	0.164009	31.0795069	0.00291491	1.80011 × 10^−26^
rs10788627	10:82203069	C	T	0.474528	15.89573196	0.00212884	0.000000016
rs1126809	11:89017961	A	G	0.302839	537.2338934	0.00231469	1 × 10^−200^
rs4578351	11:16587580	C	T	0.222048	21.90329715	0.00257235	1.69981 × 10^−15^
rs150527451	11:68817897	A	G	0.106417	10.87251081	0.00347045	4.00037 × 10^−14^
rs1437635	11:16358722	A	C	0.166029	14.4360852	0.00286686	5.19996 × 10^−13^
rs10896139	11:66650060	T	C	0.270905	12.69585036	0.00240498	1.40001 × 10^−8^
rs3213737	12:96379806	A	G	0.576	39.62292854	0.00215925	2.70023 × 10^−49^
rs1278766	13:113534382	C	T	0.545712	11.4384389	0.00213886	3.90032 × 10^−19^
rs61981034	14:97377089	A	G	0.257954	16.09645343	0.00243789	4.60002 × 10^−8^
rs3759579	14:103851272	G	A	0.588839	13.65440978	0.00215924	8.10009 × 10^−9^
rs10873552	14:105433129	G	A	0.657663	12.28285202	0.00224357	3.69999 × 10^−8^
rs11070811	15:31394082	T	C	0.183157	199.4755056	0.00274862	1.5 × 10^−10^
rs12913832	15:28365618	G	A	0.775275	19.28728164	0.00253089	1.9011 × 10^−126^
rs10220751	15:47923520	G	T	0.404814	69.33004087	0.00216389	2.5 × 10^−10^
rs11648436	16:14008674	T	C	0.356354	17.37390139	0.00221874	9.8992 × 10^−35^
rs141817469	16:89927151	T	C	0.035108	571.313217	0.00581987	1 × 10^−57^
rs1805007	16:89986117	T	C	0.100947	159.1503835	0.00350312	1 × 10^−200^
rs1805008	16:89986144	T	C	0.086593	31.32564681	0.00376737	1 × 10^−200^
rs112089506	16:90149171	T	C	0.081196	20.68388188	0.00400897	1.39991 × 10^−47^
rs1548714	17:26280204	C	A	0.811733	10.76256226	0.00272296	2.99999 × 10^−9^
rs784235	18:53423144	G	A	0.81909	10.42600087	0.0027625	2.99999 × 10^−9^
rs6059655	20:32665748	G	A	0.897978	231.9945654	0.00357982	1 × 10^−200^
rs6007506	22:45622014	T	C	0.337443	54.04116263	0.00225726	4.10015 × 10^−28^

SNP: single nucleotide polymorphism; EA: effect allele; OA: other allele; EAF: effect allele frequency.

### 3.2 Analysis of the causal effect of CSOs on melanoma

**Figure 3:**
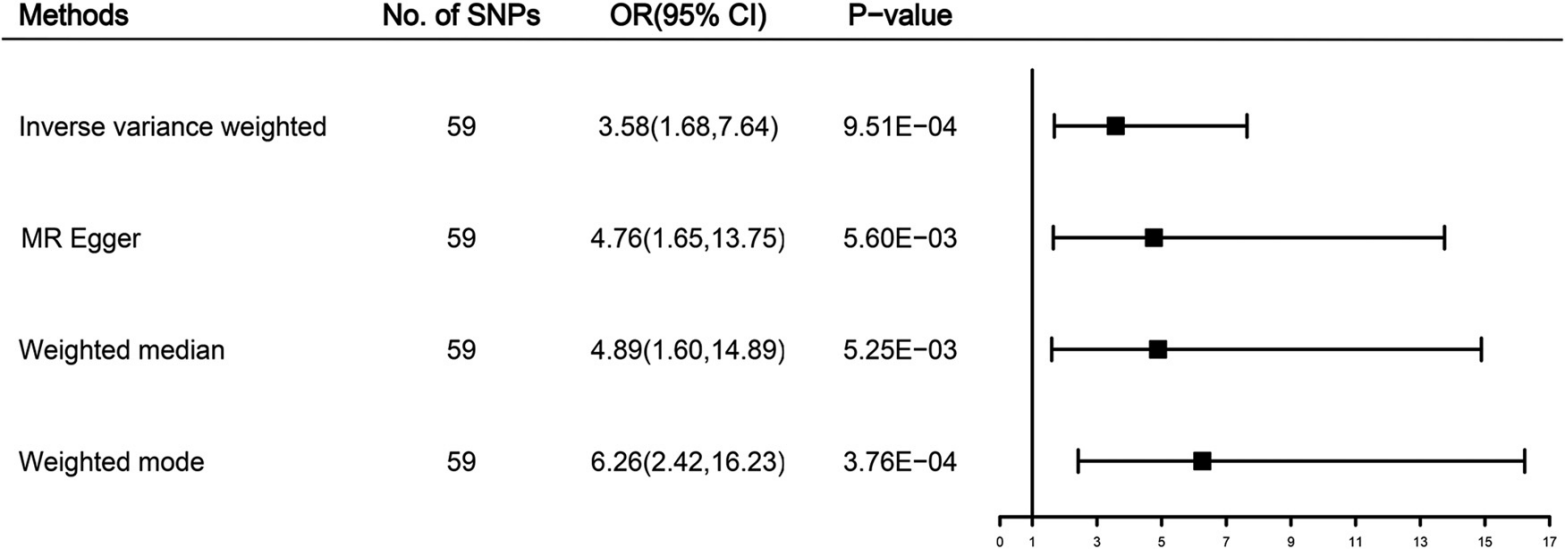
Forest plot of two-sample MR estimation of the association between childhood sunburn occasions and melanoma risk. Presented OR and CI correspond to the effects of childhood sunburn on melanoma. The results of univariable MR analyses using various analysis methods (IVW, MR-Egger, weighted median, and weighted mode) are presented for comparison. Total SNP indicates the number of genetic variants used as instruments for MR analysis.

**Figure 4:**
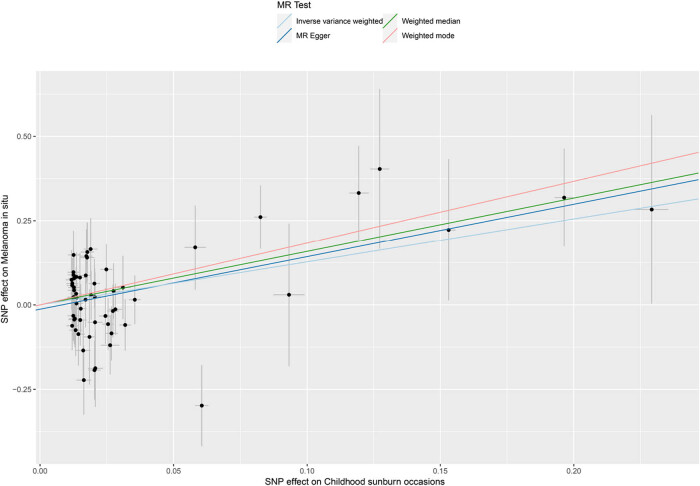
Scatter plot of SNPs associated with childhood sunburn occasions and risk on MIS. The slope of the straight line indicates the magnitude of the causal association. The light blue line represents IVW, the light green line represents weighted median, the dark blue line represents MR‐Egger, and the light pink line represents weighted mode.

When using the 59 SNPs as IVs, the random-effect IVW results showed a significant causal association between CSOs and melanoma occurrence (odds ratio [OR] = 3.58; 95% confidence interval [CI]: 1.68–7.64; *P* = 1.00 × 10^−3^). The causal association between CSOs and melanoma remained consistent across the remaining three supplements and was statistically significant. The MR-Egger (OR = 4.76, 95% CI: 1.65–13.75, *P* = 5.60 × 10^−3^), weighted median (OR = 4.89, 95% CI: 1.62–14.76, *P* = 4.90 × 10^−3^), and weighted mode (OR = 6.26, 95% CI: 2.49–15.77, *P* = 3.00 × 10^−4^) were as indicated. Additionally, MR-PRESSO did not identify significant outlier IVs, indicating the presence of no horizontal pleiotropy in this study. Figure 3 provides details of the five MR methods. The scatter plot, shown in Figure 4, demonstrates the predicted effect of IVs on CSOs and melanoma, with the increasing slope of the plot indicating a positive correlation between CSOs and the risk of melanoma occurrence.

### 3.3 Sensitivity analysis

**Figure 5:**
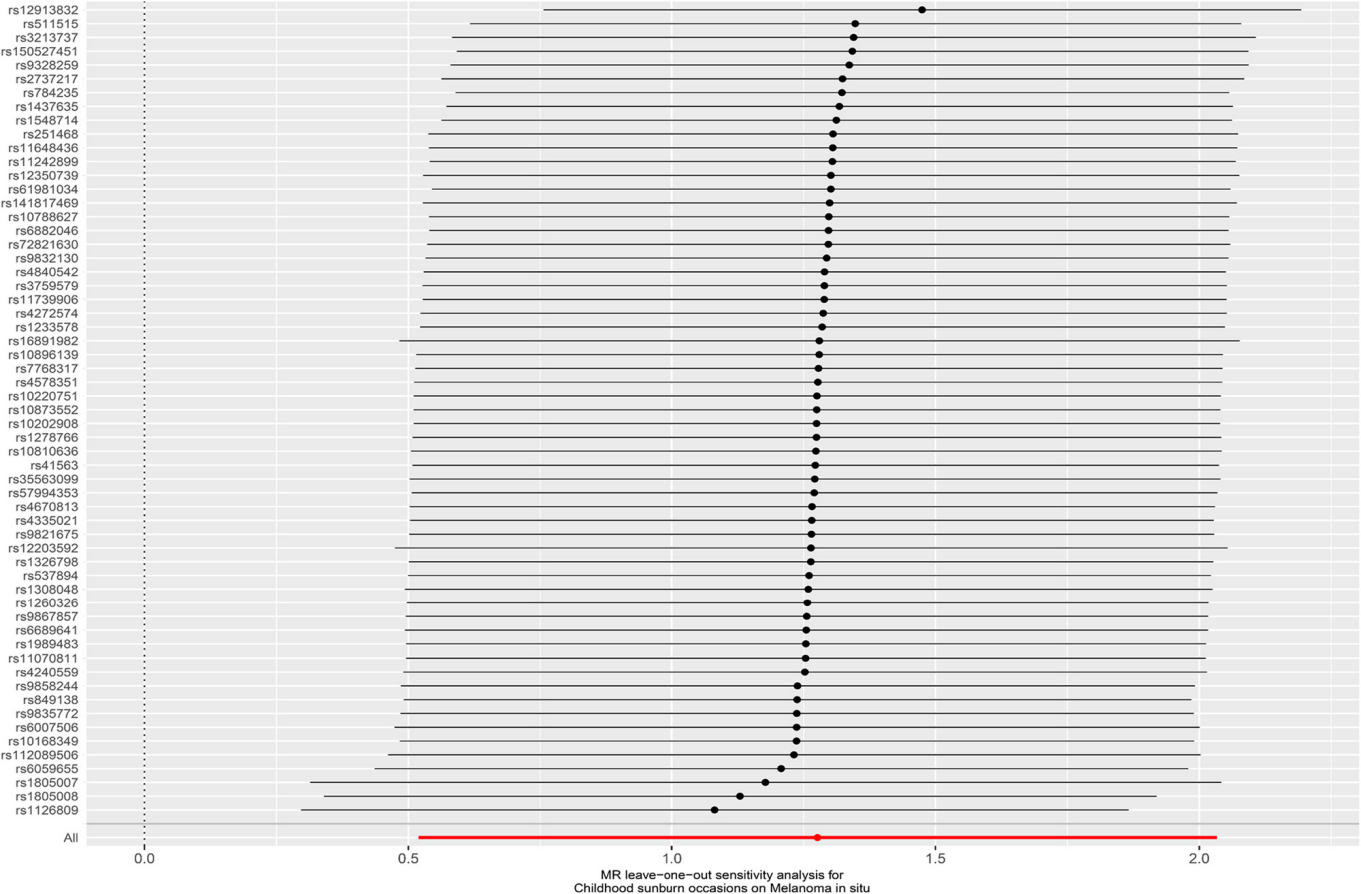
Leave-one-out analysis results of SNPs associated with childhood sunburn occasions and risk on MIS.

**Figure 6:**
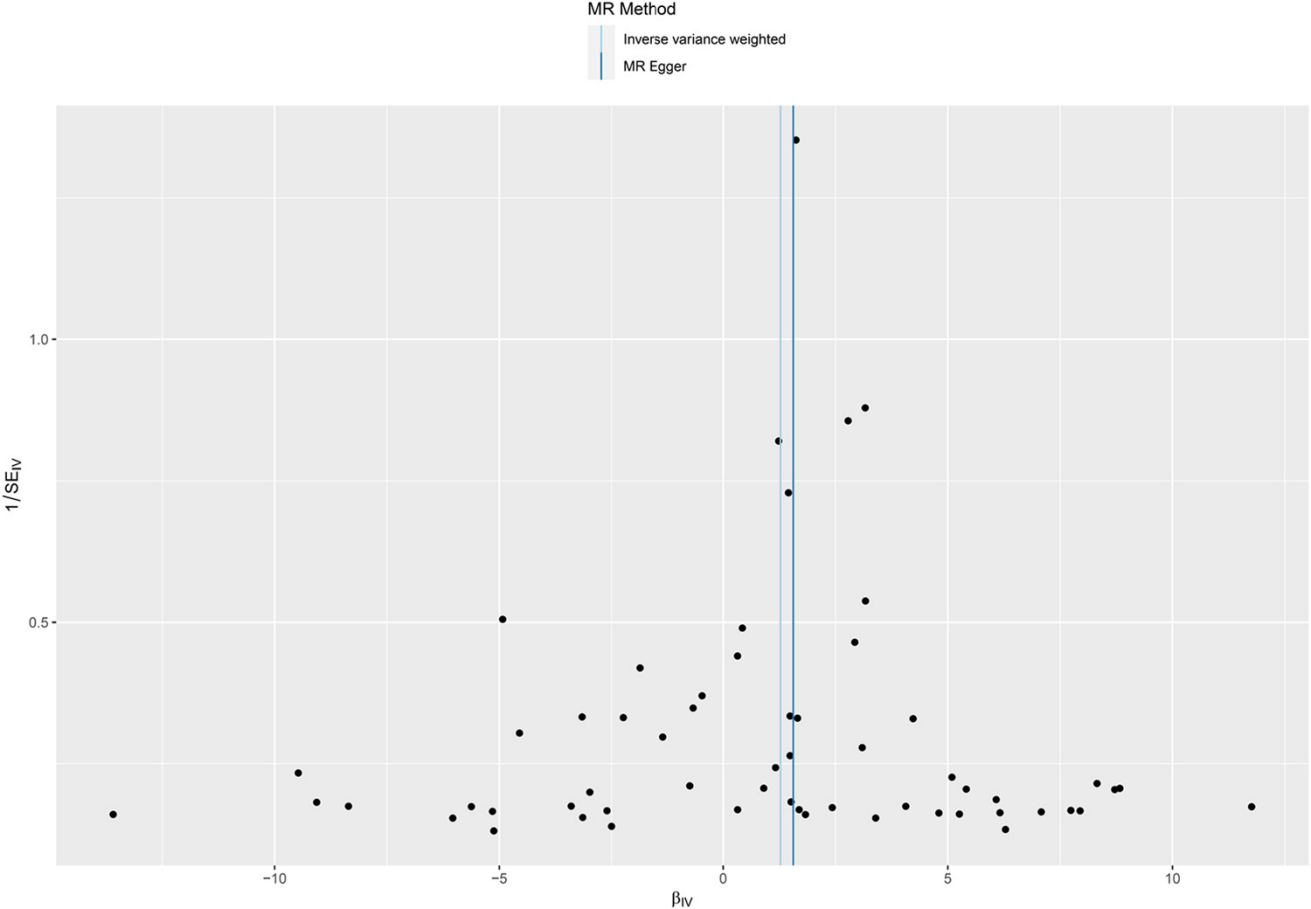
Funnel plot of relationship between the causal effect of childhood sunburn occasions on MIS and reciprocal of the SD of the causal estimation using a single SNP as an instrument.

There was no heterogeneity found in the conducted *Q* test for heterogeneity (*P* = 0.113). The MR-Egger regression method intercept significantly deviated from 0 (*P* = 0.457), suggesting the absence of horizontal pleiotropy in the IVs. The *P*-value for the MR-PRESSO global test was 0.15, indicating the absence of horizontal pleiotropy in the study. Furthermore, we found no SNP locus associated with confounding factors for outcome in the PhenoScanner_v2_ query for phenotypes or diseases associated with the SNPs included in the analysis, which were associated with phenotypes or diseases such as height, BMI, hip circumference, and years of education. The forest plot based on the LIA results shows good consistency (Figure 5). The funnel plot (Figure 6) provides the distribution and intensity of each SNP in CSOs and melanoma with good symmetry, and no significant outlier SNPs were found. These results suggest that the study results are unlikely to be influenced by potential bias.

### 3.4 Multivariate MR analysis

**Figure 7:**
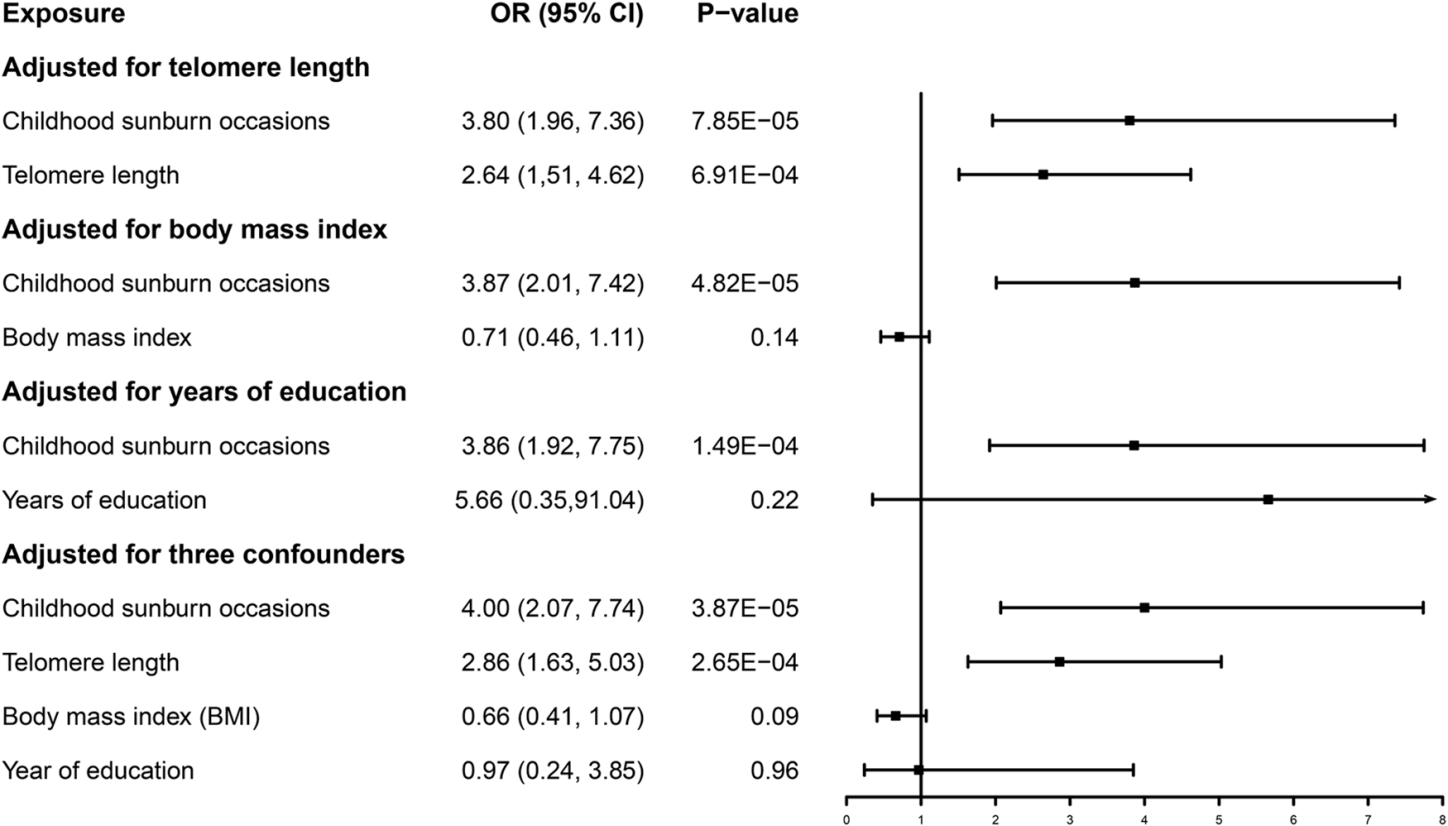
Forest plot of multivariable MR in MIS. Adjusted for telomere length, years of education, and BMI. Presented OR and CI correspond to the effects of childhood sunburn with melanoma risk.

The results of the multivariate MR analysis are shown in Figure 7. After adjusting for telomere length (OR = 3.80, 95% CI: 1.96–7.36, *P* = 7.85 × 10^−5^), BMI (OR = 3.87, 95% CI: 2.01–7.42, *P* = 4.82 × 10^−5^), years of education (OR = 3.66. 95% CI: 1.92–7.75, *P* = 1.49 × 10^−4^), and after all confounding factors (OR = 4.00, 95% CI: 2.07–7.74, *P* = 3.87 × 10^−5^), the causal association between CSOs and melanoma remained statistically significant.

## 4 Discussion

Currently, the high mortality rates associated with cancer have surpassed those associated with chronic diseases, such as cardiovascular diseases, with cancer being the leading cause of death in many countries. Melanoma, as a highly aggressive, metastatic, and poor prognosis malignant tumor, has a significantly increasing trend of morbidity and mortality despite remarkable improvements in the existing diagnosis and treatment technology. Hence, melanoma remains a great challenge to improving public health. In the era of precision medicine, the early identification of high-risk exposed populations, an in-depth understanding of melanoma risk factors and pathogenesis, and specific measures for intervention targets can effectively reduce the burden of melanoma disease.

In recent years, with the steady and orderly development of large-scale GWAS and the development of MR application model specifications, MR has made a great contribution to disease association studies. It is widely used to explore risk factors, intermediate mechanisms, and bidirectional causality among complex diseases. Furthermore, it is expected to become an important link between traditional observational studies and RCTs in the causal evidence chain. At this stage, the application of MR provides new ideas for the study of melanoma pathogenesis. It has an irreplaceable position in melanoma research. However, due to the limitations of the race of the subjects and the number of gene types, the application of MR in melanoma at this stage is limited and only stays in the etiological research, lacking pathophysiological and related translational research, which is difficult to be used for clinical practice. However, the use of MR to guide etiological research is in the ascendant, and the combination of genome-wide association analysis with multi-omics analysis represented by proteomics and metabolomics to identify disease-causing genes and potential targets for intervention may become a new way of thinking for the application of MR in melanoma.

The current study explores the causal association between CSOs and melanoma from a genetic perspective based on UK Biobank and FinnGen summary data using the TSMR approach. The results of the study showed that for every 1 SD increase in CSOs, the risk of melanoma increases by 2.58-fold in the European population. Additionally, the causal association between CSOs and melanoma remained statistically significant after controlling for confounding factors (telomere length, BMI, and years of education). This result is generally consistent with previous epidemiological studies. Chang et al. [41] included 15 case–control studies with a total of 12,916 participants to assess the risk of sunburn and melanoma development. The results showed that sunburn during childhood significantly increased the incidence of melanoma (OR = 1.5, 95% CI: 1.3–1.7). Recently, a large cohort study from Norway reported that sunburn in childhood increased the risk of melanoma [7]. Furthermore, Olsen et al. [8] conducted a prospective population-based cohort study that sought to assess the strength of the association between individual UV exposure and melanoma. A total of 15,375 participants without melanoma at baseline were included in the study, and 420 melanoma events (173 invasives, 247 *in situ*) occurred during a 4.4-year (median) follow-up period. The study showed that more than 50 sunburn experiences in childhood or adolescence significantly increased the risk of melanoma development.

As previously stated, an MR study with a rigorous design and valid core assumptions can provide reliable evidence of causal associations. First, to avoid population stratification bias, the sample resources used in this study were drawn from European populations. Additionally, 59 SNPs that were strongly associated with CSOs and independent of each other were finally included as IVs after a rigorous screening step. Meanwhile, the *F*-statistic of each SNP was greater than 10, indicating the absence of weak IVs. Second, two methods – MR-Egger regression and MR-PRESSO global test – were used to assess the horizontal multi-effectiveness of the IVs. Moreover, we used multiple statistical models, and the LIA method was used to conduct a sensitivity analysis of the main findings. These statistical methods further validated that the IVs included in the study were reasonably valid and that the study results were robust and reliable. Moreover, taking the potential confounding factors (such as telomere length, BMI, and year of education) into consideration, the genetic predisposition to childhood sunburn was still an independent risk for melanoma. Telomere length, BMI, and years of education were chosen as confounders because of the large number of studies suggesting that these factors influence tumorigenesis and development.

This is the MR study to make causal inferences about the risk of childhood sunburn events and melanoma. As always, our findings support a causal effect of childhood sunburn on the risk of melanoma development, which provides a vital public health message that raising awareness of sun protection in children and adopting necessary sun protection behaviors play a crucial role in preventing melanoma development.

## 5 Limitations

Although we achieved secondary mining of biological information in the database through innovative statistical methods, it is worth discussing the limitations of both the MR method and the GWAS database in practical applications. First, the information related to CSOs was obtained through self-reported questionnaires rather than from objective measures and hence may be subject to recall bias. Therefore, the study results need to be interpreted with caution. Second, our study population was limited to individuals of European ancestry, which effectively controlled for population stratification bias. However, whether the findings can be extrapolated to other ethnic groups, which may show differences in genetic background and lifestyle, remains to be tested. Third, it is difficult to exclude the effect of genetic pleiotropy; that is, genetic variants can affect the outcome through pathways other than “genetic variation-exposure factor-outcome.” Fourth, because we used summary data, we could not explore the non-linear effects of CSOs and melanoma [37]. In addition, this study is only a statistical result and cannot further explore the biological mechanisms between CSOs and melanoma.

## 6 Conclusion

At the genetic level, using a two-sample MR method, this study confirmed that CSOs increase the risk of melanoma development. In conclusion, avoiding UV damage, avoiding childhood sunburn, and taking precautions are key.

## Abbreviations

CSOs: childhood sunburn occasions
RCT: randomized controlled trial
GWAS: genome-wide association study
MR: Mendelian randomization
IV: instrumental variable
TSMR: two-sample Mendelian randomization
SNP: single nucleotide polymorphism
SD: standard deviation
IVW: inverse variance weighted
MR-PRESSO: Mendelian randomization pleiotropy residual sum and outlier
BMI: body mass index
EAF: effect allele frequency
OR: odds ratio
CI: confidence interval
